# Navigation-assisted surgery for chondroblastoma arising in the femoral head: A case report

**DOI:** 10.1016/j.ijscr.2020.03.049

**Published:** 2020-04-19

**Authors:** Takanori Maru, Jungo Imanishi, Tomoaki Torigoe, Kazuo Saita, Yuho Kadono, Yasuo Yazawa

**Affiliations:** a Department of Orthopaedic Oncology and Surgery, Saitama Medical University International Medical Center, Japan; b Department of Orthopaedic Surgery, Saitama Medical Center, Saitama Medical University, Japan; c Department of Orthopaedic Surgery, Saitama Medical University Hospital, Japan

**Keywords:** Chondroblastoma, Femoral head, Navigation, Curettage, Surgery, Case report

## Abstract

**Introduction:**

Surgery for chondroblastoma in the femoral head is challenging due to its inaccessibility, with high risk of local recurrence and poor functional outcomes reported. We herein report the first case of chondroblastoma in the femoral head treated by navigation-assisted surgery.

**Presentation of case:**

A 12-year-old girl presented with persistent left hip pain and limited hip range of motion. Imaging studies revealed a well-defined osteolytic lesion in the left femoral head accompanied by extensive intra-osseous oedematous change. The bone lesion was radiologically diagnosed as chondroblastoma. With navigation assistance, curettage was performed via the anterior approach. The tumor was fully accessible from the femoral neck. After curettage, the bony defect was filled with bone substitute. The pathological diagnosis was chondroblastoma. The post-operative course was uneventful. Thirty months postoperatively, the patient was free of pain with full hip range of motion, and MR images showed no evidence of recurrence or osteonecrosis.

**Discussion:**

This case is the first to use a navigation system for the treatment of chondroblastoma in the femoral head. The navigation system can minimize damage to intact structures and increase the efficiency of curettage by visualizing access to the tumor.

**Conclusion:**

Navigation assistance is an optimal surgical option for chondroblastoma in the femoral head.

## Highlights

• We reported the first case to use navigation for the femoral head chondroblastoma.
• Visualization of tumor on navigation helps to minimize unnecessary destruction.
• Navigation assistance is an optimal surgical option for chondroblastoma in the femoral head.

## 1 Introduction

Chondroblastoma is a rare benign bone tumor, accounting for 1–2% of primary bone tumors. It usually affects the epiphysis or apophasis of the long bones in adolescents [1]. The femoral head is the fourth most common site for chondroblastoma, with its incidence being 5–8% of all chondroblastomas [2,3]. Despite its rarity, chondroblastoma of the femoral head has been a topic of debate because of the high risk of recurrence and osteoarthritis [[2], [3], [4], [5], [6], [7]]. There are major issues associated with its anatomical features; the femoral head is rounded, deep seated, and is connected to the femoral shaft by the narrow femoral neck. Some approaches may be inappropriate for specific tumor locations. The inaccessibility of the tumor also means a higher risk of incomplete tumor removal and recurrence. Another issue is the vascularity of the femoral head. Osteonecrosis is a potential major post-operative complication of chondroblastoma in the femoral head, often requiring further surgical intervention such as osteotomy and total hip replacement [4,6,7]. Minimal invasiveness and preservation of the vascularity of the femoral head are important.

The navigation system can guide surgeons to allow more accurate interventions such as osteotomy and screw insertion. This technology has become a standard treatment for spine, trauma and joint surgeries [[8], [9], [10]], and is gaining popularity for tumor surgeries [11,12]. For chondroblastoma in the femoral head, where precise removal of the tumor and minimal damage to the bony and vascular structures are required, the navigation system can be a solution strategy. We herein report the details of the surgery and clinical outcomes following navigation-assisted curettage of a femoral head chondroblastoma in an adolescent. This surgery was conducted at a university hospital, and this work has been reported in line with the SCARE criteria [13].

## 2 Presentation of case

**Fig. 1:**
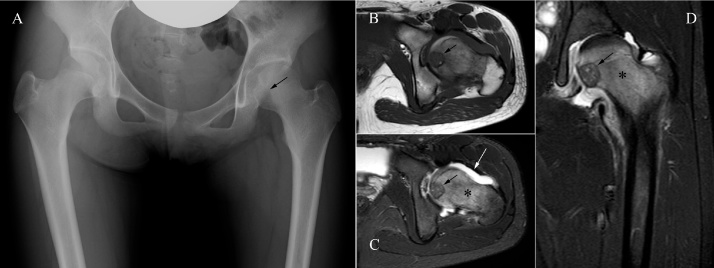
A radiograph (A) and MR images (B–D) at first presentation; B: T1-weighted axial image; C: STIR axial image; D: STIR coronal image. The tumor was located just underneath the joint cartilage (black arrows), and was surrounded by extensive oedema (*) and accompanied by hip joint effusion (white arrow).

A 12-year-old girl presented with a three-month history of left hip pain and limited hip range of motion. Radiographic studies revealed a well-defined osteolytic lesion of 2 cm in diameter in the femoral head with joint effusion and extensive oedema around the lesion (Fig. 1). Based on its typical tumor location and radiologic features, this lesion was diagnosed as chondroblastoma, and we planned to perform surgery without biopsy.

**Fig. 2:**
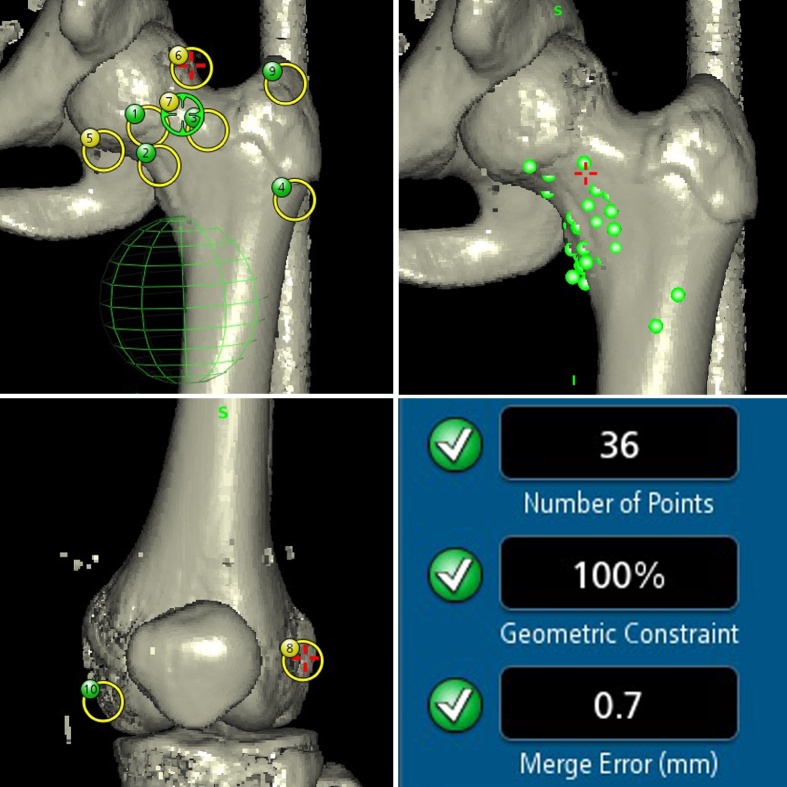
Paired registration using 10 points including the distal portion of the femur, surface marking of 36 points, and the results of registration.

**Fig. 3:**
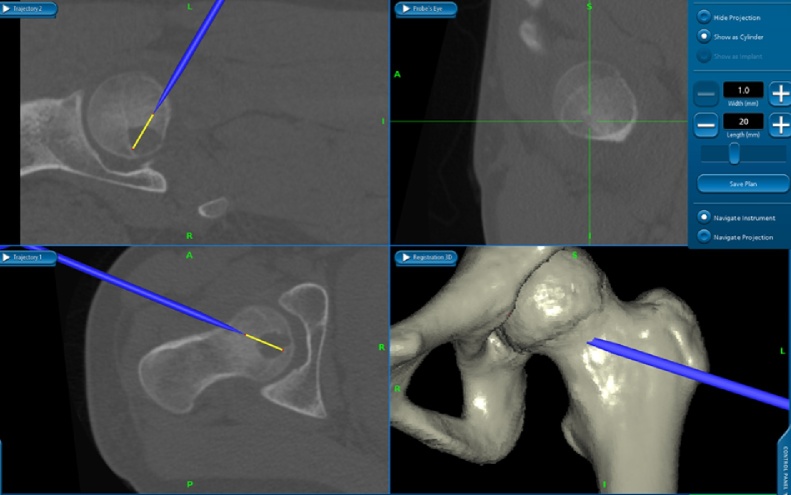
Intraoperative navigation monitor showing the shortest route to the tumor. The blue needle-like object represents the tracker, and the yellow line shows the distance to the tumor.

**Fig. 4:**
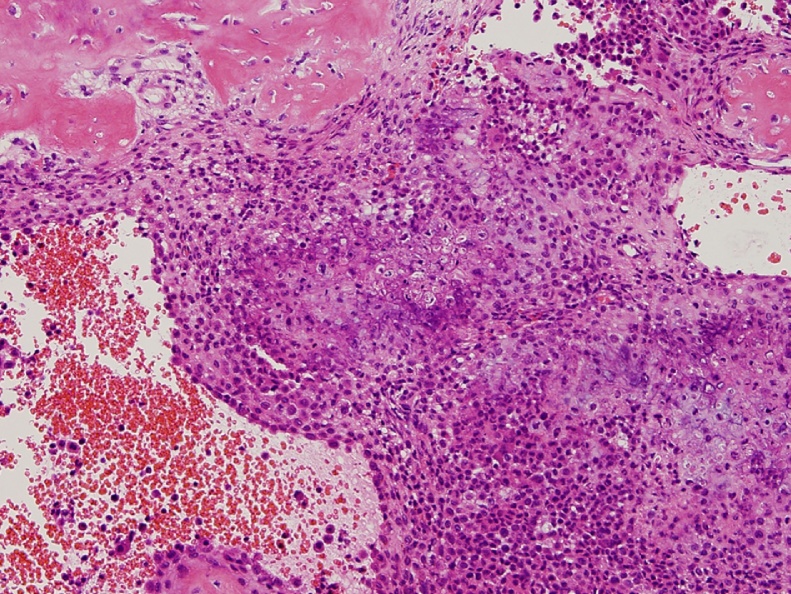
Hematoxylin and eosin staining of the curetted specimen.

**Fig. 5:**
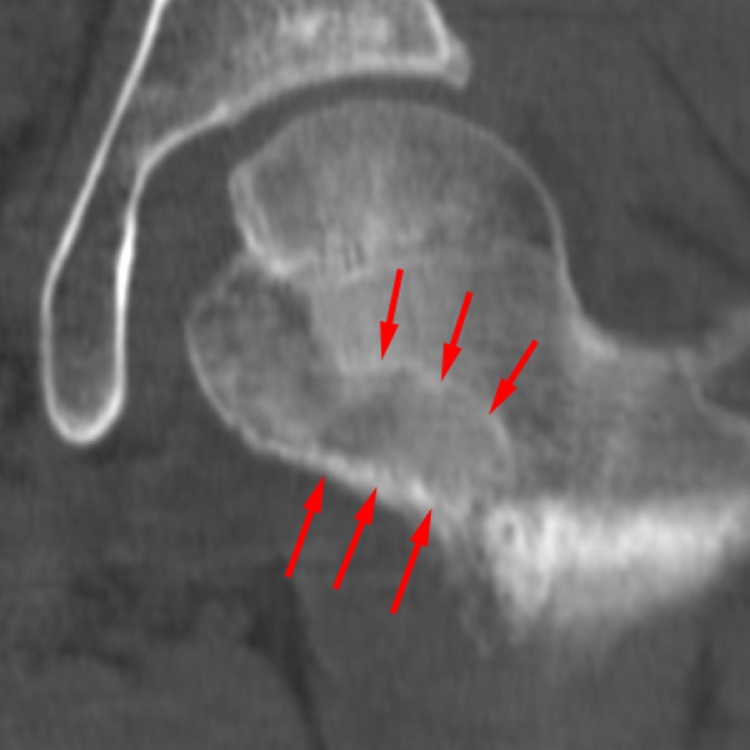
A reconstructed CT image at two weeks post-surgery. Red arrows show the route taken to reach the tumor.

The surgery was performed by a board-certified orthopaedic surgeon (JI), with 15 years of experience in orthopaedics. The tumor was curetted via the anterior hip approach (Smith-Petersen approach). The tumor was invisible on C-arm imaging. The lateral femoral circumflex artery was identified and preserved. The navigation system used in our case was the S7 Stealth Station (Medtronic Inc., Littleton, MA, USA). Helical CT DICOM data for the whole left femur were used for intra-operative navigation. Two reference pins were inserted in the proximal shaft of the femur, and the tracker was fixed to the pins. The exact points for the paired registration points were identified by C-arm imaging together with visual inspection and palpation. Initially, we attempted to complete paired registration using proximal femoral landmarks only, but failed. Two distal femoral landmarks were added for paired registration. After paired registration and 36 surface markings, we were able to obtain accuracy of 0.7 mm (Fig. 2). The navigation system revealed that the tumor was accessible from anterior femoral neck with the left hip in 20 degrees of internal rotation (Fig. 3). A 1-cm round window was made in the anterior femoral neck cortex to afford an approach to the tumor, and then cancellous bone was minimally removed to allow access to the tumor with navigation assistance. After curettage, the defect was filled with hydroxyapatite/collagen bone-like nanocomposite (HAp/Col). The pathologic diagnosis of the curetted specimen was chondroblastoma (Fig. 4). CT images at two weeks after surgery showed that the route taken to the tumor was the shortest possible (Fig. 5).

**Fig. 6:**
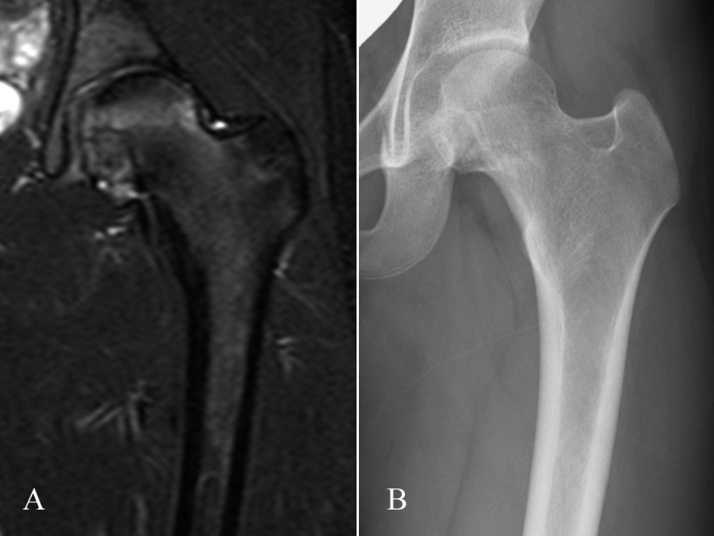
A STIR MR image at 18 months after surgery (A) and a radiograph at 31 months after surgery (B).

The post-operative course was uneventful. One-quarter weight bearing (PWB) for the first 4 weeks, followed by 1/2 PWB for 4 weeks and then full weight bearing was permitted for the left lower limb. Six months post-operatively, the patient resumed dancing, which was her main hobby, and remained free of pain or limited hip range of motion. At 31 months after surgery, the patient remained asymptomatic and did not show any limitation to hip range of motion or limb length discrepancy, with a full MSTS score [14]. The latest MR images showed no evidence of recurrence, and the extensive oedema and hip joint effusion had disappeared. The latest radiograph showed bone regeneration in the defect after tumor removal (Fig. 6).

## 3 Discussion

Chondroblastoma occurs mostly at the epiphyses or apophyses of the long bones in adolescents and young adults and causes pain [1]. Standard treatment is surgical curettage and bone grafting, with local recurrence rates ranging from 14 to 18% in the literature [2,3,6]. Due to its unique location, being in proximity to the joint, and its tendency to affect adolescents, care must be taken to minimize damage to the joint and growth plate.

For chondroblastomas in the femoral head, four treatment options have been published: (1) curettage via the sub-greater trochanter, (2) curettage through the anterior femoral neck, (3) resection via the hip joint with hip dislocation, and (4) CT-guided radio frequency ablation [[2], [3], [4],^15^,^16^]. However, all the aforementioned options have their specific pros and cons. The risk of fracture is high in the first and second options, whereas hip joint degeneration can occur more often in association with the third option. In addition, the surgical approach should be chosen carefully in consideration of tumor location as the accessible zones differ among these procedures.

Six reports on chondroblastoma of the femoral head were summarized (Table 1). Although bias in the level of proficiency of the surgeon and the approach used should be considered, these reports revealed that local recurrence, hip arthritis and femoral head necrosis were not infrequent. Three studies reported high local recurrence rates of 20–67% with no femoral head necrosis or hip osteoarthritis [2,5,6], whereas two studies reported less frequent local recurrence but a total of five patients required total hip replacement [4,7]. This contrast may indicate an inverse correlation between local recurrence and femoral head necrosis/hip osteoarthritis: wider curettage/resection may prevent local recurrence but increase the risk of femoral head necrosis/hip osteoarthritis. Visualization of the tumor itself and an approach utilizing the shortest route to the tumor can help to reduce unnecessary bone destruction in gaining access to the tumor. In our case, the use of navigation assistance allowed precise access to the tumor with minimal invasiveness.

**Table 1:** Past literature related to chondroblastomas in the femoral head.

Study (Year)	Procedure	No.	Follow-up duration	Local recurrence	Hip surgery	MSTS score [14]
Ramappa (2000) [6]	Not stated	8	2–12 years	4/8 (50%)	Osteotomy (1 case)	Not available
Lin (2005) [3]	Curettage via anterior femoral neck	4	1–23 years	0/4 (0%)	None	Not available
Xu (2014) [4]	Modified Trapdoor	13	3–9 years	0/13 (0%)	THA (1 case)	93–100%
Hapa (2016) [5]	Curettage (different approaches)	3	1–9 years	2/3 (67%)	None	57–70%
Farfalli (2017) [7]	Curettage (different approaches)	8	2–17 years	1/8 (13%)	THA (4 cases)	Mean, 80%
Leitinen (2019) [2]	Mixed (incl. 5 RFAs)	14	1–15 years	3/14 (21%)	None	Not available

*MSTS* Musculoskeletal Tumor Society.

Navigation-assisted surgery for bone tumor resection has gained in popularity since it was first reported in 2004 [17,18]; however, some pitfalls related to navigation assistance should be considered. In a recent retrospective study of 78 cases undergoing navigation assisted resection of bone tumors by Farfalli et al., they reported 5% registration failure where they had to continue surgeries without navigation assistance [19]. Takao et al. analyzed seven cases undergoing proximal femoral osteotomy using CT-based navigation, and insisted on the importance of understanding the error range associated with the navigation-guided procedure [20]. For bone tumor resection with navigation assistance, the total discrepancy of the cutting point; i.e., “positional error”, between the pre-operative planning and actual procedure includes navigation error and cutting error. The former has been reported to be up to 1 mm in most cases [12,19], whereas the latter can be around 1 mm considering the width of the bone saw. This positional error, along with angle error, may have to be considered if the tumor is very small and far from the bone surface, but such small errors within 2 mm can be ignored for the majority of bone tumors.

## 4 Conclusion

We report the first case of chondroblastoma in the femoral head successfully treated with navigation-assisted curettage. The medium-term clinical outcomes were satisfactory, indicating that the navigation system affords a promising option for chondroblastoma in the femoral head.

## Declaration of Competing Interest

None.

## Funding

None.

## Ethical approval

Ethical board approval is not required for case reports in our institute.

## Consent

The patient and her mother were informed that clinical information including radiographs, photographs and videos from the case would be submitted for publication and gave their written consent on 27 January 2020. A copy of the written consent is available for review by the Editor-in-Chief of this journal on request.

## Registration of research studies

1. Name of the registry: (Not Applicable).
2. Unique identifying number or registration ID: (Not Applicable).
3. Hyperlink to your specific registration (must be publicly accessible and will be checked): (Not Applicable).

## Guarantor

Mr. Jungo Imanishi.

## Provenance and peer review

Not commissioned, externally peer-reviewed.

## CRediT authorship contribution statement

**Takanori Maru:** Investigation, Data curation, Writing - original draft. **Jungo Imanishi:** Conceptualization, Investigation, Resources, Writing - review & editing, Supervision. **Tomoaki Torigoe:** Writing - review & editing. **Kazuo Saita:** Writing - review & editing. **Yuho Kadono:** Writing - review & editing. **Yasuo Yazawa:** Writing - review & editing.
